# Assessment of Dust, Chemical, Microbiological Pollutions and Microclimatic Parameters of Indoor Air in Sports Facilities

**DOI:** 10.3390/ijerph20021551

**Published:** 2023-01-14

**Authors:** Justyna Szulc, Robert Cichowicz, Michał Gutarowski, Małgorzata Okrasa, Beata Gutarowska

**Affiliations:** 1 Department of Environmental Biotechnology, Faculty of Biotechnology and Food Science, Lodz University of Technology, Wólczańska 171/173, 90-530 Łódź, Poland; 2 Institute of Environmental Engineering and Building Installations, Faculty of Civil Engineering, Architecture and Environmental Engineering, Lodz University of Technology, Al. Politechniki 6, 90-924 Łódź, Poland; 3 Department of Personal Protective Equipment, Central Institute for Labour Protection—National Research Institute, 90-133 Łódź, Poland

**Keywords:** air quality, sports facilities, microclimatic parameters, particulate matter, chemical contamination, bioaerosol, biodiversity

## Abstract

The aim of this study was to analyse the quality of indoor air in sport facilities in one of the sport centres in Poland with respect to microclimatic parameters (temperature, humidity, and air flow velocity), particulate matter concentrations (PM_10_, PM_4_, PM_2.5_, and PM_1_), gas concentrations (oxygen, ozone, hydrogen sulphide, sulphur dioxide, volatile organic compounds, and benzopyrene), and microbial contamination (the total number of bacteria, specifically staphylococci, including *Staphylococcus aureus*, haemolytic bacteria, *Enterobacteriaceae*, *Pseudomonas fluorescens*, actinomycetes, and the total number of fungi and xerophilic fungi). Measurements were made three times in May 2022 at 28 sampling points in 5 different sporting areas (the climbing wall, swimming pool, swimming pool changing room, and basketball and badminton courts) depending on the time of day (morning or afternoon) and on the outside building. The obtained results were compared with the standards for air quality in sports facilities. The air temperature (21–31 °C) was at the upper limit of thermal comfort, while the air humidity (RH < 40%) in the sports halls in most of the locations was below demanded values. The values for dust pollution in all rooms, except the swimming pool, exceeded the permissible limits, especially in the afternoons. Climatic conditions correlated with a high concentration of dust in the indoor air. Particulate matter concentrations of all fractions exceeded the WHO guidelines in all researched premises; the largest exceedances of standards occurred for PM_2.5_ (five-fold) and for PM_10_ (two-fold). There were no exceedances of gaseous pollutant concentrations in the air, except for benzopyrene, which resulted from the influence of the outside air. The total number of bacteria (5.1 × 10^1^–2.0 × 10^4^ CFU m^−3^) and fungi (3.0 × 10^1^–3.75 × 10^2^ CFU m^−3^) was exceeded in the changing room and the climbing wall hall. An increased number of staphylococci in the afternoon was associated with a large number of people training. The increased concentration of xerophilic fungi in the air correlated with the high dust content and low air humidity. Along with the increase in the number of users in the afternoon and their activities, the concentration of dust (several times) and microorganisms (1–2 log) in the air increased by several times and 1–2 log, respectively. The present study indicates which air quality parameters should be monitored and provides guidelines on how to increase the comfort of those who practice sports and work in sports facilities.

## 1. Introduction

Today, a healthy lifestyle based on regular physical activity is widespread worldwide. The World Health Organisation (WHO) recommends that adults undertake at least 2.5 h of moderately intense activity per week [1]. With the increasing number of sports enthusiasts, there is a growing number of indoor sports facilities with climbing walls, swimming pools, tennis courts, and full-size playing fields, etc., in one building [1].

Indoor air quality in sports facilities is an important factor that influences the comfort of those exercising, the work of coaches and referees, and the performances of athletes [1,2,3,4]. It also affects the health of the users and employees of sports facilities. Specifically, the amount of inhaled pollutants increases proportionally to increasing lung ventilation rates. This means that during exercise most of the air is inhaled through the mouth, bypassing the normal nasal mechanisms for filtering large particles and soluble vapours, with the increased airflow velocity carrying pollutants deeper into the airways [5]. Indoor air pollution may therefore increase the risk of irritation, allergic sensitisation, acute and chronic respiratory disorders, and the impairment of lung function [6].

Air quality in sports facilities depends on a number of factors including the temperature, humidity, air velocity, and concentrations of particulate matter (PM) and the presence of bioaerosols, CO_2_ and other gases, and volatile organic compounds (VOCs) [7].

Air temperature is the most perceptible parameter for users of sports facilities, and it also has the greatest influence on thermal comfort [8]. A sports room should be maintained at a temperature that ensures heat dissipation without creating cold or hot sensations [9]. The American Society of Heating, Refrigerating, and Air-Conditioning Engineers (ASHRAE) recommends that temperature and humidity should be maintained within comfort limits during the summer season in the ranges of 21–24 °C at RH = 30% or temperatures of 20–23 °C at higher RH = 60%; the dynamics in terms of changes in these parameters should not exceed 20% per hour [2].

The air velocity in the occupied zone is also an important factor in thermal comfort parameters. If it is too high, it causes a feeling of coldness, draughts, and dust rising from the floors; if it is too low, it can inefficiently remove heat from the skin surface, thus increasing the feeling of suffocation, heat, and, consequently, sweating [10]. The recommended maximum values according to ASHRAE are 0.15 m s^−1^ for sports fields and 0.2 m s^−1^ for indoor sports halls.

Indoor air quality is affected by the particulate matter present, with this being divided into fine particles (PM_2.5_) and coarse particles (PM in the range 2.5–10 μm). The sub-fraction of PM_2.5_ examined is the submicron fraction (PM_1_). Due to the varying ability of PM to move and be deposited in the respiratory tract, PM is divided into three fractions [11]: 1. inhalation fraction-particles with aerodynamic diameter ≤100 μm, which are retained in the upper respiratory tract and do not enter the alveoli; 2. trachea fraction-particles < 30 μm, which pass into the trachea during breathing; and 3. respirable fraction-particles < 4 μm, which can settle in the alveoli and pass into physiological fluids. Respirable particulate matter deposited on the alveolar walls can cause difficulties in gas exchange, meaning that under conditions of high PM concentrations, athletes will tire more quickly and their performance will be reduced by up to 3–5%, which can also affect training and competition performance [12,13,14]. The effects of airborne particulate matter on human health can vary depending on its physical properties (size and shape, surface area, electrical charge, and hygroscopicity) and properties related to light scattering and absorption as well as the chemical properties of the dust (its elemental composition and the presence of inorganic ions and organic compounds) [3,15].

A reference for good room ventilation is the concentration of carbon monoxide and carbon dioxide in the air. The carbon monoxide in sports facilities studied to date has only been present at low or undetectable levels, never exceeding the WHO 2010 guidelines (15 min-100 ppm, 1 h-35 ppm, 24 h-7 ppm). Similarly, CO_2_ concentrations have not exceeded 1000 ppm [2].

An important marker of indoor air quality that can affect the health of people in sports facilities is the concentration and type of microorganisms in the bioaerosol of such facilities. The literature indicates the presence of mannitol-positive staphylococci in the air of sports rooms, including antibiotic-resistant species, such as *Staphylococcus epidermidis, S. hominis, S. capitis, S. sciuri, S. xylosus, S. warneri,* and *S. haemoliticus*, and fungi of the genera *Cladosporium, Penicillium, Fusarium, Acremonium,* and *Alternaria* [16].

It important to remember that the purpose of sports premises determines the key air quality parameters. Indoor climbing walls are specific rooms. Athletes using them use magnesia (magnesium carbonate MgCO_3_ or magnesium hydroxycarbonate MgCO_3_ × Mg(OH)_2_ × H_2_O) to increase grip, which can be a source of PM_1_, PM_2.5_, and PM_10_ in the air of such facilities [17,18]. The health risks of long-term exposure to inhaled magnesia are evidenced by Moshammer et al.’s study of 109 individuals before and after climbing activity, who were found to have adverse acute and subacute lung symptoms after physical activity [19].

Studies of indoor air at indoor swimming pools and aquatic activity centres indicate that, in this sporting environment, chemical compounds used for water disinfection are the greatest air pollution problem [20]. Air testing for microbiological contaminants in swimming pools is very rarely performed, in contrast to water testing. In Italy, Brandi et al. investigated the occurrence of fungi in the air in 10 pools and found low levels of contamination with high biodiversity [21]. Mould species of the genera *Penicillium* spp., *Aspergillus* spp., *Cladosporium* spp., *Alternaria* spp., *Fusarium* spp. were consistently detected in the air samples of the pools and represented the typical microflora of outdoor air, with *Candida albicans* yeast not being detected. In Iran, Mansoorian et al. found the presence of the following bacteria in the air of swimming pools: *Escherichia coli*, *Pseudomonas alcaligenes*, *Pseudomonas aeruginosa*, *Klebsiella pneumonia*, and actinomycetes; contamination with these bacteria was observed in roughly 17% of samples [22].

Studies on the assessment of sports hall air quality indicate that the most commonly examined parameters in these premises are oxygen–carbon dioxide concentration, temperature, and relative humidity [23,24,25].

On the other hand, the microbiological contamination of sports hall air was examined by Onet [26] and independently by Ilies [27]. The total count of bacteria in the air of sports halls was in the range of 0–6.9 × 10^3^ cfu m^−3^, while the total count of fungi was 0–1.6 × 10^3^ cfu m^−3^. It was found that haemolytic bacteria, which are an indicator of pathogenic microorganisms, were present in high concentrations at intensive sporting activities. In addition, a positive correlation between microorganisms (fungi and bacteria) and the number of people indicated that intense sports activity influences the variability of microorganisms in the air of these premises [26,27].

Despite indoor air quality being important and the fact that the number of professional multisport facilities is increasing, there is still an insufficient number of analyses of this type of building in Poland and worldwide. There is a lack of information on the key parameters determining indoor air quality with specificity related to dedicated sports.

The aim of the study was to analyse the indoor air quality in the sports activity facilities (the climbing wall, swimming pool, changing room at the swimming pool, basketball/volleyball courts, and badminton court) of one multisport centre in a major city in Central Europe, the Sports Centre of the Łódź University of Technology (Zatoka Sportu, Łódź, Poland).

The analysis took into account the following parameters in the air: microclimate (temperature, humidity, and flow velocity), gaseous pollutants (oxygen, ozone, hydrogen sulphide, sulphur dioxide, and volatile organic compounds, including benzopyrene), particulate matter (PM_10_, PM_4_, PM_2.5_, and PM_1_), and the microbiological contamination of surfaces in the examined rooms (the total count of bacteria and fungi, including xerophilic fungi, mannitol-positive staphylococci, *Staphylococcus aureus*, haemolytic staphylococci, *Enterobacteriaceae, Pseudomonas fluorescens*, and actinomycetes). In addition, the microbial biodiversity of the air was also assessed using molecular biology methods (high-throughput DNA sequencing). Pollutions were examined in terms of sports activity (in the morning and in the afternoon, where the number of people practicing sports was 5–10 times higher) and depending on the type of activity.

The current research is the first such extensive analysis of the quality of the environment of a sports building and a pioneering presentation of the results of a metagenomic analysis of the air for this type of premises.

## 2. Materials and Methods

### 2.1. Study Site and Sampling Strategy

**Figure 1:**
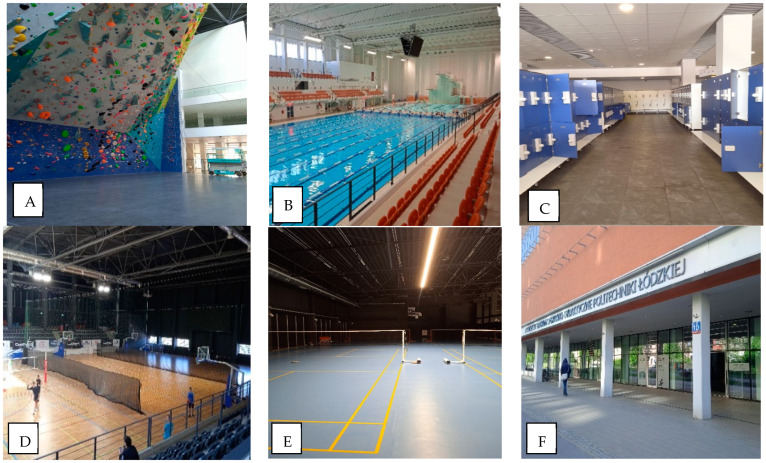
Analysed locations: (**A**) “climbing wall”, (**B**) “swimming pool”, (**C**) “changing room at the swimming pool” (**D**) “basketball/volleyball court”, (**E**) “badminton court”, and (**F**) “entrance to the building”.

Measurements were taken in 5 different facilities of the sports centre (the climbing wall, swimming pool, changing room, basketball court, and badminton court) in 28 sampling points and in the atmospheric air outside the building (Table 1, Figure 1). The sports facility is the Academic Sports and Didactic Centre of the Technical University of Lodz, Zatoka Sportu, which was established in 2016 and hosts individual and group sports training and mass events such as the European Academic Games and Academic Sports Championships. The volume of the facility is 262,518 m^3^, and the height of the building is 22 m; the length and width of the facility are 171.2 m and 69.7 m; the number of storeys is 4/1 (overground/underground); and the number of seats in the auditorium is 4327. Regarding the power supply system for air-handling unit heaters in the administrative, social, and sports sections, the calculated thermal power of the installation is 625.62 kW, the total power transferred by the installation is 661.951 kW, the power supply system for heaters serving the swimming pool and the total power transferred by the installation is 459.57 kW, and the total power from the ventilation module is 1121.521 kW.

The rooms are ventilated using a mechanical supply and exhaust ventilation system. The exhaust is located under the roof/ceiling, while the air supply corresponds to the specific construction requirements of a given room, e.g., in the swimming pool, there are air supply slots (0.075 m^2^) in the floor space and below the seats in some of the stands. The ventilation devices for pitch rooms and walls are supply and exhaust units with filtration, heating, and humidification sections. In the case of the swimming pool hall and the locker room, a set of air handling units are equipped with filtration, humidification, drying, heating, and cooling sections. The main entrance to the building overlooks a busy street.

A study of microbiological air contamination was carried out for each analysed location in several repetitions and during three days with an interval of one week (on the 5 May 2022, 12 May 2022, and 19 May 2022). The measurements for each sampling day were performed twice: in the morning prior to the commencement of sports activities and in the afternoon during the most intensive use of the sports facilities (Table 1). At the same time, the microclimate and particulate matter concentration as well as chemical contamination were analysed. Moreover, the microbial contamination of the surfaces in the analysed facilities was determined (Table 1). Additionally, samples of internal and external air were taken to determine the biodiversity of microorganisms in bioaerosols.

### 2.2. Analysis of Microclimate Parameters

The temperature, relative humidity, and air velocity were measured at each site. The air velocity was measured three times at an interval of 3 min at each location in the morning and afternoon using an ECOM-DPH anemometer at a height of 2 m from the floor. The relative humidity (RH) was measured with a TES-1360 digital humidity meter (RH measurement range: 10–95%, accuracy ±3%). The temperature was measured with a DT-34 digital thermometer (measurement range −50 °C to +270 °C, accuracy 0.8 °C) at a distance of at least 1.5 m from human concentrations.

### 2.3. Analysis of Particulate Matter Concentration

The particulate matter (PM) concentration was measured using a DustTrak™ DRX Aerosol Monitor 8533 portable laser photometer (TSI, Dallas, TX, USA). The device simultaneously measured the mass concentrations corresponding to the fractions PM_2.5_, PM_4_, and PM_10_ and respirable and total dust. The detection range for particles with diameters ranging from 0.1 to 15 μm was between 0.001 and 150 mg m^−3^. The measurements were obtained in triplicate for each location at the height of 1.5 m from ground level. The sampling rate was set at 3 L min^−1^ and the sampling interval at 5 s. The total sampling time was 3 min.

### 2.4. Analysis of Chemical Air Contamination

Chemical air pollution analyses were performed for the sampling locations, specifically the climbing wall (A), basketball/volleyball court (D), badminton court (E), and entrance to the building (F), while due to the high humidity that could damage the sensors, tests were not performed for the swimming pool (B) and the swimming pool’s changing room (C). For the analysis, a piece of portable multi-sensor equipment was used, which is a module composed of the following sensors: a Scentroid DR1000, for the determination of VOC and O_3_ concentrations; an EcoWatch with a metal oxide semiconductor (MOS) detector, which measures the concentration of total VOCs (ethanol, isobutane, benzo-α-pyrene, 0–500 ppm); and an electrochemical (EC) detector to measure the concentrations of O_2_ (0.20–100%), H_2_S (3 ppb–1 ppm), SO_2_ (0.5–2000 ppm).

### 2.5. Determination of Airborne Microorganism Number

Volumes of 100 L of air were collected in triplicate (at a height of about 1.5 m from ground level) using a DUO SAS Super 360 (VWR International Ltd., Milan, Italy) according to CEN EN 13,098 [28]. Atmospheric air (external background) was also collected in front of the building. The microbiological contamination of the air was determined using the following media: TSA (Tryptic Soy Agar: Merck, Darmstadt, Germany) with (0.2%) nystatin, for the determination of the number of bacteria; MEA (Malt Extract Agar, Merck, Germany) medium with (0.1%) chloramphenicol—fungi; DG18 LAB-AGAR™ (DG18 Agar, Biocorp, Poland)—xerophilic fungi; Chapman Agar with (0.2%) nystatin—mannitol-positive Staphylococcus spp. (Merck, Germany); Baird Parker Agar (Merck, Germany) with (0.2%) nystatin (Merck, Germany)—*Staphylococcus aureus*); Columbia Blood Agar with (0.2%) nystatin (BTL, Warsaw, Poland)—haemolytic Staphylococcus; King B medium with (0.2%) nystatin, (Merck, Germany)—*Pseudomonas fluorescens*; Violet Red Bile Glucose Agar (VRBG LAB-AGAR) with (0.2%) nystatin (Biocorp, Warszawa, Poland)—*Enterobacteriaceae*; and Pochon’s agar with (0.2%) nystatin (Labomix, Łódź, Poland)—actinomycetes.

The samples were incubated depending on the type of microorganisms in the following conditions: temp. 37 ± 2 °C for 24–48 h (for mannitol-positive and mannitol-negative staphylococci, *Staphylococcus aureus, Enterobacteriaceae*, and haemolytic bacteria); temp. 30 ± 2 °C for 48 h (for the total number of bacteria and *Pseudomonas fluorescens*); and temp. 25 ± 2 °C for 5–7 days (for the total number of fungi, xerophilic fungi, and actinomycetes). After incubation, the colonies were counted, and the results (arithmetic mean) were expressed in CFU m^−3^.

### 2.6. Determination of Surface Microbial Contamination

Samples from surfaces were taken using Hygicult^®^ TPC (Orion Diagnostica Oy, Finland) with the total plate count medium. Samples were taken from four–five locations in each of the sport facilities (Table 1) in three independent repetitions and incubated at temp. 30 °C ± 2 °C for 2–5 days. Next, the colonies were counted, and the results (arithmetic mean) were expressed in CFU 100 cm^−2^.

### 2.7. Determination of Biodiversity

In order to determine the biodiversity of microorganisms in the air of the sports facilities and the control, air samples from the climbing wall hall and outside the building were taken. Air (8000 L) was passed through sterile gelatine filters (80 mm, 0.3 µL Sartorius, Göttingen, Germany) using AirPort MD 8 (Sartorius, Germany). The analysis was performed in accordance with the methodology described by Szulc et al., [29]. In brief, genomic DNA was extracted using Genomic Mini AX Bacteria + set (A&A Biotechnology, Gdańsk, Poland) with additional mechanical lysis with zirconium oxide beads of the samples (FastPrep-24). Next, the DNA was treated using an Anti-Inhibitor Kit (A&A Biotechnology, Poland). The DNA concentration was 2–30 µg mL^−1^ (real-time PCR). In the reaction, universal primers amplifying the 16S rRNA bacterial gene’s fragment and fungal ITS regions were used [30,31]. The libraries of V3–V4 and ITS amplicons were prepared by Macrogen (Seoul, Republic of Korea) according to the guidelines of 16S Metagenomic Sequencing Library Preparation Part # 15044223 Rev. B. For the two-step PCR, the Herculase II Fusion DNA Polymerase Nextera XT Index Kit V2 was used.

The sequencing was performed using the paired-end technology on Illumina MiSeq (2 × 300 bp) platform by Macrogen (Republic of Korea).

Bioinformatic analysis of the obtained sequencing for taxonomic identification was performed according to Jensen et al. [32]. Bioinformatic analysis was carried out using CLC Genomic Workbench v. 12 (Qiagen, Hilden, Germany) + Microbial Genomics Module Plugin v. 4.1 (Qiagen).

### 2.8. Statistical Analysis

Statistical analysis was carried out with Statistica 13.1 (Statsoft, Tulsa, OK, USA). Descriptive statistics were calculated for all variables of interest. The normality of the distribution was checked using the Shapiro–Wilk and Levene tests at a significance level of 0.05.

In the case of normally distributed data, means were compared between the analysed locations using a one-way analysis of variance (ANOVA) at a significance level of 0.05. The ANOVA assumptions were checked with the Levene tests. When a statistical difference was detected (*p* < 0.05), the means were compared using Tukey’s post-hoc procedure at a significance level of 0.05. In the case of non-normally distributed data, Kruskal–Wallis tests followed by Dunn’s posthoc tests at the significance level of 0.05 were performed.

## 3. Results and Discussion

### 3.1. Microclimate Parameters

**Figure 2:**
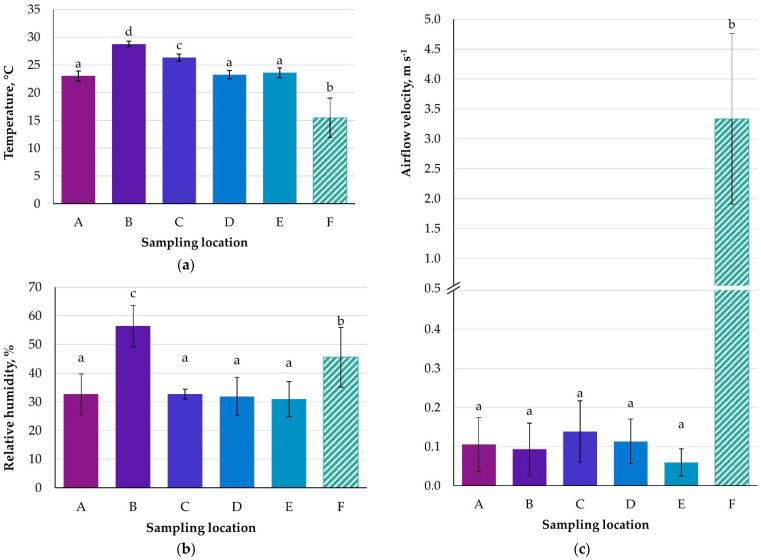
Bar chart showing averaged microclimate conditions at the selected locations; (**a**) temperature, (**b**) relative humidity, (**c**) airflow velocity; means marked with different lowercase letters (a−d) within the particular parameter are significantly different; ANOVA, *p* < 0.05; Tukey’s HSD test, *p* < 0.05. (A) “climbing wall”; (B) “swimming pool”; (C) “changing room at the swimming pool”; (D) “basketball/volleyball court”; (E) “badminton court”; (F) “entrance to the building”.

The atmospheric air temperature was 11.9 °C–20.0 °C, depending on the day of measurement and in the premises of the centre ranged from 21.0 °C–29.5 °C. The highest temperature occurred in the swimming pool, with lower values being recorded in the changing room (25.6 °C–27.1 °C). In the remaining locations, the temperature range was 21.5 °C–24.7 °C (Table S1; Figure 2a). Statistically significantly higher temperatures (*p* < 0.05) were observed in the swimming pool (B) and the changing room (C) when compared to the other premises. Meanwhile, the atmospheric air in front of the building had a significantly lower temperature (*p* < 0.05) (Figure 2a).

According to the Polish standard PN-78/B-03421, the temperature in rooms with high physical activity should be 15 °C–18 °C in winter and 18 °C–21 °C in summer [33]. Comparing the obtained results with this standard, we can find that they are at the upper end of the range or even exceed it by 2 °C–3 °C, meaning they could affect thermal comfort. The ASHRAE [34] recommends a temperature of 20 °C for a general sports hall. For facilities for specific sports, one can use the guidelines of the Badminton World Federation for badminton [35] and of the Fédération Internationale de Basketball for the basketball court [36]. The guidelines envisage a temperature range of 18 °C–30 °C for badminton and a range of 16 °C–20 °C for basketball, respectively. According to the measurements, the temperature on the badminton courts varied between 22 °C–25 °C, which is within these recommendations, while on the basketball court the measured values were always above 22 °C, which is above the FIBA’s guidelines.

In the swimming pool, the air temperature value is related to the water temperature in the basin in accordance with the technical conditions for the pool hall air conditioning system. In order to reduce water evaporation, the heaters warm the supplied air to the pool hall so as to maintain a 2 °C higher air temperature in relation to that of water. This approach is in line with studies by Sabiniak and Kolaszewski [37,38]. The world swimming federation (FINA) is also in agreement in this regard. In the swimming pool’s changing room, according to PN-78/B-03421, the temperature should not exceed 24 °C, while in the examined room the temperature was found to exceed this by 2–3 degrees depending on the time of day.

The relative humidity was similar in the examined locations (23.8–44.2%), with the exception of the swimming pool area, where the highest values, of almost 65%, were recorded (*p* < 0.05). The atmospheric air humidity was statistically significantly different (*p* < 0.05) from the indoor locations (excluding the swimming pool) and ranged from 32.1–54.8% (Table S1; Figure 2b).

Analysing the relative humidity values in the sports premises, it can be concluded that this parameter was below the demanded values, not exceeding 40%, given as the minimum [33]. In several measurements, the humidity in the sports premises was far too low, with reports from the building personnel that they felt dry mucous membranes and increased dustiness. On the basketball court, the humidity was within the range of the FIBA recommendations, which state an RH < 50% but do not specify a minimum limit for this parameter [36].

In all rooms, similar values in terms of air velocity were recorded (0.0 m s^−1^–0.2 m s^−1^), differing significantly from the values obtained outside the facility (2.0 m s^−1^–4.4 m s^−1^) (Table S1; Figure 2c).

According to the Polish standard PN-78/B-03421, to ensure comfort in a sports hall, the maximum air velocity should not exceed 0.3 m s^−1^ in winter and 0.5 m s^−1^ in summer [33]. Higher values may cause a feeling of draught and cold. The measurement results obtained are within these limits. ASHRAE recommends that the velocity should be less than 0.25 m s^−1^ [34], while a different approach is represented by Steimle, who defines lower and upper limits of 0.18–0.41 m s^−1^ [39]. Taking into account the increased temperature in the room, it would appear to be best to set the velocity in the occupied zone at 0.15 m s^−1^ in order to balance it. On the badminton court, where, following the guidelines of the BWF federation, the maximum velocity should be 0. 2 m s^−1^ (due to the use of light darts in this sport) [35], it was found that the measured velocity in the examined room did not exceed 0.1 m s^−1^. The world federation FIBA has not established guidelines for air velocity on basketball courts, and higher values were achieved in the afternoon, with a maximum value of 0.17 m s^−1^ [36]. The adopted general values of standards for sports halls classify this result as acceptable. In the case of the swimming pool hall, the most stringent recommendations give a figure of 0.15 m s^−1^ as the limit value in the occupied zone [37,40], and the measured values exceeded the recommended value.

### 3.2. Oxygen Concentration Measurement and Assessment of Chemical Air Pollution

**Figure 3:**
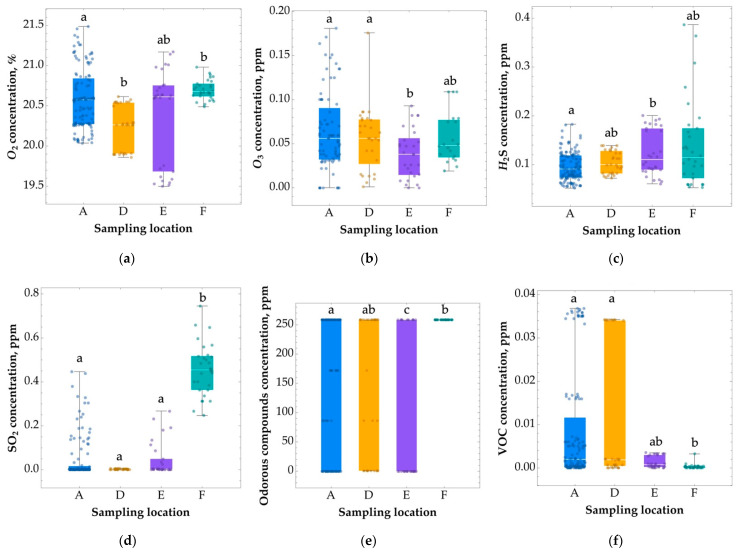
Box and whisker plots showing the averaged oxygen and chemical contaminant concentrations at the selected locations. The whiskers represent the minimum and maximum values of all the data. Results with different letters (a–c) are significantly different from each other (Kruskal–Wallis test followed by Dunn’s posthoc tests at a significance level of 0.05). (A) “climbing wall”; (D) “basketball/volleyball court”; (E) “badminton court”; (F) “entrance to the building”. VOC—benzopyrene determined using an EcoWatch sensor with a metal oxide semiconductor detector (no other compounds were detected).

The range of variation in oxygen concentration in the indoor air of the sports premises was 19.6–21.2% (Table S2, Figure 3a). The average O_2_ concentration in the sports facility during the research period was 20.5%. A statistically significant lower (*p* < 0.05) oxygen concentration was recorded in room D (the basketball/volleyball court). However, changes in this range are imperceptible to the human body.

The concentration of hydrogen sulphide in the air at the examined locations remained in the range of 0.06–0.24 μg m^−3^ (Table S2, Figure 3c). The H_2_S concentration in the air was not exceeded at any of the measurement points (TLV—threshold limit value: 7 μg m^−3^) according to the Polish Regulation of the Minister of Family, Labour and Social Policy [41].

The highest ozone concentration occurred at the climbing wall and averaged 0.11 μg m^−3^ (Table S2, Figure 3b). The lowest averaged O_3_ concentration at the whole site was 0.04 μg m^−3^. These values are well below the normative limits, and it can be concluded that, during the study, the ozone concentration was not exceeded at any point (TLV: 0.14 μg m^−3^) according to the above-mentioned regulation.

Sulphur dioxide concentrations were measured in the atmospheric air and in the examined rooms. SO_2_ was detected in the greatest concentrations (*p* < 0.05) in the atmospheric air, with concentrations ranging from 0.34 ppm to 0.53 ppm (Table S2, Figure 3d). The indoor air limit (0.5 ppm) set by the WHO (2010) was not exceeded in any of the examined rooms [42].

The measurement of VOCs in the air showed the presence of these compounds at low concentrations, in the range of 0–0.04 ppm (Table S2, Figure 3e). The study detected low concentrations of benzo-α-pyrene, which belongs to VOCs. The concentration of benzo-α-pyrene in the climbing wall hall was 4.5 μg m^−3^ (Table S2, Figure 3f). At the same time, elevated levels of benzo-α-pyrene were recorded in the air outdoors, which is directly connected to the climbing wall through the entrance door of the facility, which is itself placed near the avenue, with a high level of traffic, with this being the likely cause of the penetration of the pollutant into the facility. The measured level of benzo-α-pyrene exceeds the permissible concentration (2 μg m^−3^ over an 8-h measurement time) by more than two times according to the Polish Regulation of the Minister of Family, Labour and Social Policy [43]. It is a particularly toxic and carcinogenic component of smoke, having the ability to accumulate in the body (Holnicki et al., 2022). In Poland, the concentration of benzo-α-pyrene in the air increasingly exceeds normative values by several times [44].

### 3.3. Particulate Matter Concentration

**Figure 4:**
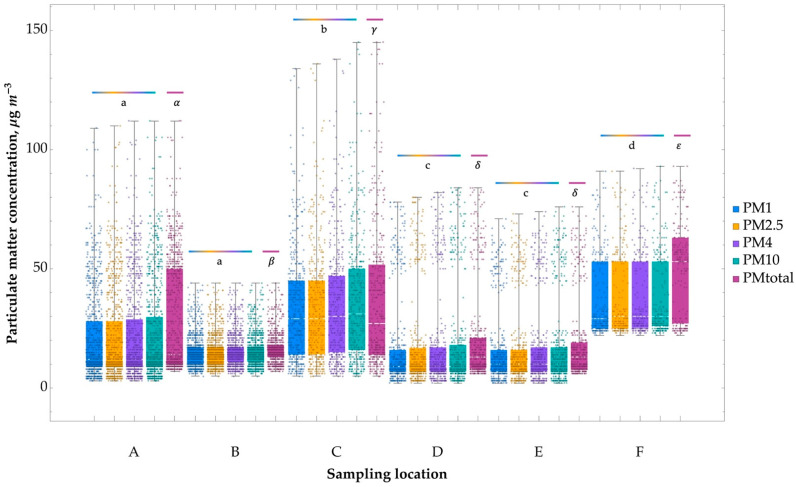
Box and whisker plot showing the particulate matter (PM) concentration at the selected locations. The whiskers represent the minimum and maximum values. Results with letters a–d (for PM_1_−PM_10_) and symbols αβγδε (for PM_total_) are significantly different from each other (Kruskal–Wallis test followed by Dunn’s posthoc tests at a significance level of 0.05). (A) “climbing wall”; (B) “swimming pool”; (C) “changing room at the swimming pool”; (D) “basketball/volleyball court”; (E) “badminton court”; (F) “entrance to the building”.

In the present research, airborne dust with dimensions below 1 µm (PM_1_) was found to be dominant (99.65–99.99% of total dust). The lowest concentrations among the analysed locations were observed in the basketball/volleyball court (D) and badminton court (E), while the highest was measured in the changing room (C) next to the climbing wall. Significant differences were observed between all indoor locations and the control air outside the facility (Figure 4).

According to EU legislation, the annual average concentration of dust with dimensions below 2.5 µm PM_2.5_ (i.e., the fraction containing the PM_1_ fraction) should not exceed 25 µg m^−3^ [45]. In our case, the measured PM_2.5_ concentration was more than twice as high as the environmental threshold, regardless of the sampling site. This agrees with the literature suggesting that the quality of air inside training facilities is often worse than outdoors [2,46].

In addition, regular exceedances of the WHO standard values for PM_10_ and PM_2.5_ concentrations were observed in the climbing wall hall and in the swimming pool changing room (0.06–0.07 mg m^−3^) during the afternoon, when most people use the facility. In the changing room, the maximum value was very high and amounted to PM_2.5_ = 0.09 mg m^−3^. Additionally, in the basketball and badminton courts, PM_2.5_ = 0.06 mg m^−3^ values exceeding the permissible limits in the afternoon. The increased dustiness corresponds to the intensity of human use of the premises and the movement of air, with particles being caused by people playing sports. Elevated dustiness in the air next to the climbing wall may result from the use of magnesia during training. As demonstrated by Weinbruch et al. and Alves et al. in 2014, magnesia can be a source of PM_1_, PM_2.5_, and PM_10_ in the air of climbing walls [17,18]. Moreover, people using indoor sports facilities may be exposed to particulate matter from a range of sources, including human-generated particles (e.g., sloughed-off skin cells, hair, and clothing fibres), biological particles (e.g., bacterial and fungal cells), bits of dead insects, soil particles, pollen, microplastics, and metal and oxide particles from nano-enhanced products, such as health and fitness products. All of those are likely to lead to inhalation exposure in humans, depending mainly on the particle size and composition. Scientific evidence shows that particles with high proportions of elemental or organic carbon or high oxidative potential might be more damaging to pulmonary health [47]. Additionally, certain PM components can interact with each other, as in the case of nanoparticles and semi-volatile organic compounds, possibly causing mixed aggregates exposure [48,49].

The presence of airborne particulate matter can affect the health of users and decrease their physical performance by around 5% [12,14].

Studies show that exposure to high concentrations of PM can increase the risk of suffering from various respiratory and circulatory diseases [50,51]. Moreover, some studies suggest that people who regularly exercise are more prone to experiencing the effects of air pollution than those who do not participate in sports [52,53].

Respirable particulate matter (particles < 4 μm) deposited on the walls of the alveoli can cause difficulties in gas exchange, meaning that under conditions of high PM concentrations, athletes will tire more quickly and their performance will be reduced by up to 3–5%, which can also affect training and performance [12,13,14].

Previous research has shown that the high number of people who exercise at closed sports facilities can contribute to air quality issues inside them. The factors that determine the intensity of exercise are also known to affect air quality [54]. The concentration of fine particles in the air inside a facility is known to fluctuate depending on the weather conditions. Furthermore, such facilities’ ventilation and air filtration systems play an important role in proper air exchange and purification.

### 3.4. Determination of Number of Microorganism in the Air and on Surfaces

**Figure 5:**
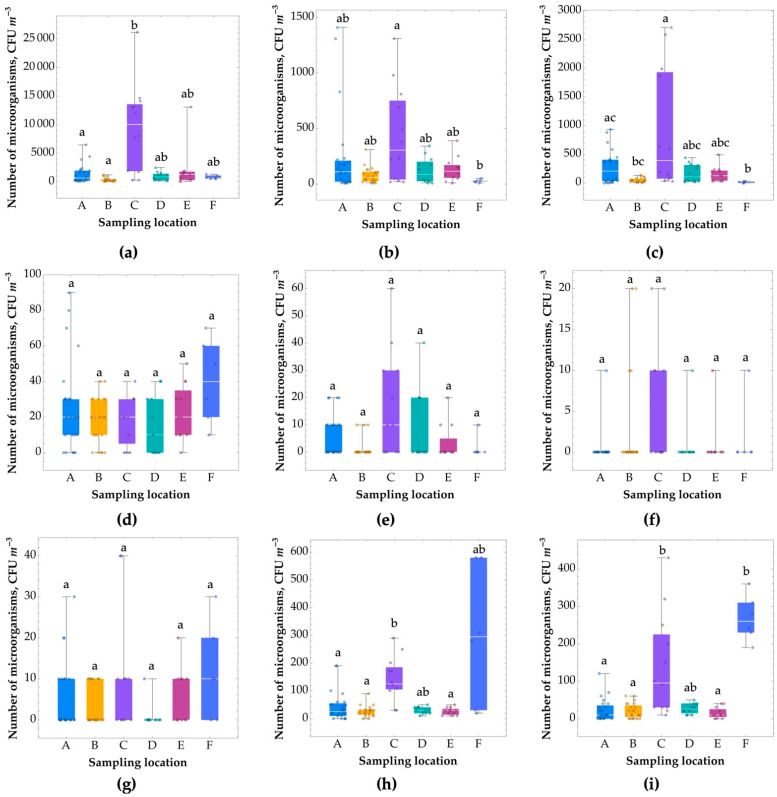
Boxplot showing the microorganism numbers at the selected locations; (**a**) bacteria, (**b**) *Staphylococcus* spp., (**c**) *Staphylococcus aureus*, (**d**) hemolizujące, (**e**) *Enterobacteriaceae*, (**f**) *Pseudomonas fluorescens*, (**g**) promieniowce, (**h**) fungi, (**i**) xerophilic fungi. The ends of the boxplot whiskers represent the minimum and maximum values of all the data. Results with different letters (a–c) are significantly different from each other (Kruskal−Wallis test followed by Dunn’s posthoc tests at a significance level of 0.05). (A) “climbing wall”; (B) “swimming pool”; (C) “changing room at the swimming pool”; (D) “basketball/volleyball court”; (E) “badminton court”; (F) “entrance to the building”.

A higher total bacterial count (5.1 × 10^1^–2.0 × 10^4^ CFU m^−3^) than in the atmospheric air (5.35 × 10^2^–1.2 × 10^3^ CFU m^−3^) was found in the examined sports premises (Table S3, Figure 5a). An exceedance of the limits of the Expert Panel on Biological Agents of the Interdepartmental Committee on Occupational Exposure Limits [55] for mesophilic bacteria (<5.0 × 10^3^ CFU m^−3^) was found for four sites: the climbing wall, changing room, swimming pool, and badminton court. In addition, elevated levels of bacteria in the air were found on the basketball/volleyball court.

For all the sports premises, there was a correlation between a higher number of bacteria in the air (by one order of magnitude) and afternoons, when sporting activity was high and the number of people playing sport significantly exceeded the number of people present in the morning. Moreover, the movement of people and the type of sports activity had an impact on the degree of bacterial air pollution. On the climbing wall, the increased number of bacteria in the air could be related not only to the higher number of people but also a high dust concentration (dust can be a carrier for bacteria). The basketball and badminton courts were used in the afternoons, which caused increased indoor air movement. Similarly, in the changing room, the intensive movement of a large number of people caused air movement, which promoted the floating of bacteria in the form of bioaerosol.

Human-derived bacteria of the genus *Staphylococcus* sp., including *S. aureus* species, were determined in the air (Table S3, Figure 5b,c). Mesophilic bacteria limits were not exceeded for *Staphylococcus* sp. (including *S. aureus*) in the air in any of the rooms examined. The highest number of *S. aureus* bacteria in the air occurred in the afternoon in the changing room of the swimming pool (6.1 × 10^2^–2.4 × 10^3^ CFU m^−3)^ and next to the climbing wall (3.8 × 10^1^–3.1 × 10^2^ CFU m^−3^). Elevated levels of these bacteria in the air were also recorded in the afternoon on the basketball/volleyball and badminton courts (1.6 × 10^2^–4.0 × 10^2^ CFU m^−3^). The high number of staphylococci in the air was closely related to the high number of people staying/playing sports in the rooms surveyed and may pose a health risk.

The number of haemolytic bacteria (of pathogenic potential) in the air was low (0–7.5 × 10^1^ CFU m^−3^) (Table S3, Figure 5d), and it did not exceed neither the literature recommendations for mesophilous bacteria nor the WHO guidelines for haemolytic bacteria (1 × 10^2^ CFU m^−3^). Similarly, the number of *Enterobacteriaceae* in the air, which is an indicator of faecal environmental pollution, was at a low level (0–3.0 × 10^1^ CFU m^−3^) (Table S3, Figure 5e) not exceeding the acceptable recommendations, which indicates good hygienic conditions. The highest number of *Enterobacteriaceae* bacteria was found in the air of the changing rooms, on the basketball court, and next to the climbing wall in the afternoon. In the rooms surveyed, the number of *Pseudomonas fluorescens* bacteria in the air was the lowest among the microbial groups surveyed (0–2.0 × 10^1^ CFU m^−3^), and in many rooms these microorganisms were not detected in the air (Table S3, Figure 5f). Only in the air of the changing rooms was this bacterium detected.

The sports facilities were also surveyed for the number of actinomycetes and fungi, which are indicative of environmental pollution. Closely related to the presence of particulate matter in the air, they originate from the soil and float on dust particles in the form of bioaerosols. In the sports rooms, the number of actinomycetes was low (0–4.0 × 10^1^ CFU m^−3^), not exceeding the acceptable recommendations, indicating good sanitary conditions. The highest number of actinomycetes was detected in the atmospheric air and in the air of the changing rooms, next to the climbing wall, and on the badminton court (Table S3, Figure 5g). The highest numbers of fungi (including xerophilic fungi) were found in the atmospheric air (2.5 × 10^1^–3.0 × 10^2^ CFU m^−3^), in the changing room air (3.0 × 10^1^–3.75 × 10^2^ CFU m^−3^), and next to the climbing wall (5.0 × 10^0^–1.0 × 10^2^ CFU m^−3^) (Table S3, Figure 5h,i). The elevated fungal count in these rooms may be related to higher dust concentrations and atmospheric air effects. The levels of fungal contamination in the air of the investigated rooms did not exceed the recommendations for fungi (<5.0 × 10^3^ CFU m^−3^) established by the Expert Panel on Biological Agents of the Interministerial Committee on Occupational Exposure Limits [55], but they did exceed the WHO recommendations (1.5 × 10^2^ CFU m^−3^) [42].

In the case of fungi, the occurrence of a higher number of microbes in the indoor air in the afternoon did not show a clear correlation. Their number depended mainly on the type of premises and the day of sampling and was rather due to the influence of outside environmental conditions.

It is worth noting that the present research has shown higher microbiological air contamination than previously published studies conducted in sports facilities (for instance, gyms, fitness rooms, and sports halls) in other countries in Europe (Poland, Italy, Romania, Czech Republic), where lower microbial contamination was observed for bacteria (5.80 × 10^1^ to 1.02 × 10^3^ CFU m^−3^) and fungi (2.10 × 10^1^ to 1.44 × 10^2^ CFU m^−3^) [16,26,54,56,57,58,59]. The elevated number of microorganisms in the air was linked with the high amount of people exercising in the afternoon and the specifics of the sport practiced.

The count of bacteria and fungi on selected surfaces in the sports bay area is shown in Figure S1. The count of bacteria on the surfaces determined in the morning was 4.0 × 100 CFU 100 cm^−2^ (the mirror in the changing room)–3.7 × 10^3^ CFU 100 cm^−2^ (the seating area in the swimming pool). For samples taken in the afternoon, the count was higher: 1.2 × 10^2^ CFU 100 cm^−2^ (the paper towel dispenser in the changing room) to 4.56 × 10^3^ CFU 100 cm^−2^ (the seating area in the swimming pool). There was a low level of fungal contamination concerning the examined surfaces, from 0 to 5.62 × 10^2^ CFU 100 cm^−2^. Fungi were most abundant on: the starting post (swimming pool), the cover for the basketball basket structure, the seat in the stands (basketball court), and a seat for the followers (tennis court). Little research has been published to date on the assessment of the microbial contamination of the surfaces in sports facilities. Boonrattanakij et al. (2021) checked the number of bacteria on sports equipment, specifically a bicycle handle, a dumbbell, and a sit-up bench (3.90 × 10^4^–3.72 × 10^5^ CFU 100 cm^−2^). In the present study, lower microbiological contamination of surfaces was detected due to changing habits (disinfecting hands and equipment regularly) among users and employees of sports facilities to prevent the spread of COVID-19 [60].

### 3.5. Diversity of Bioaerosols

The present research is the first detailed report on the biodiversity of airborne microorganisms in sports facilities. The advantage of metagenome analysis is the identification of microorganisms directly from the test sample, skipping the cultivation stage, which prevents the loss of species of microorganisms that are non-culturable under laboratory conditions [61,62]. This method was therefore successfully used to asses biodiversity of various kinds of environmental samples [63].

**Figure 6:**
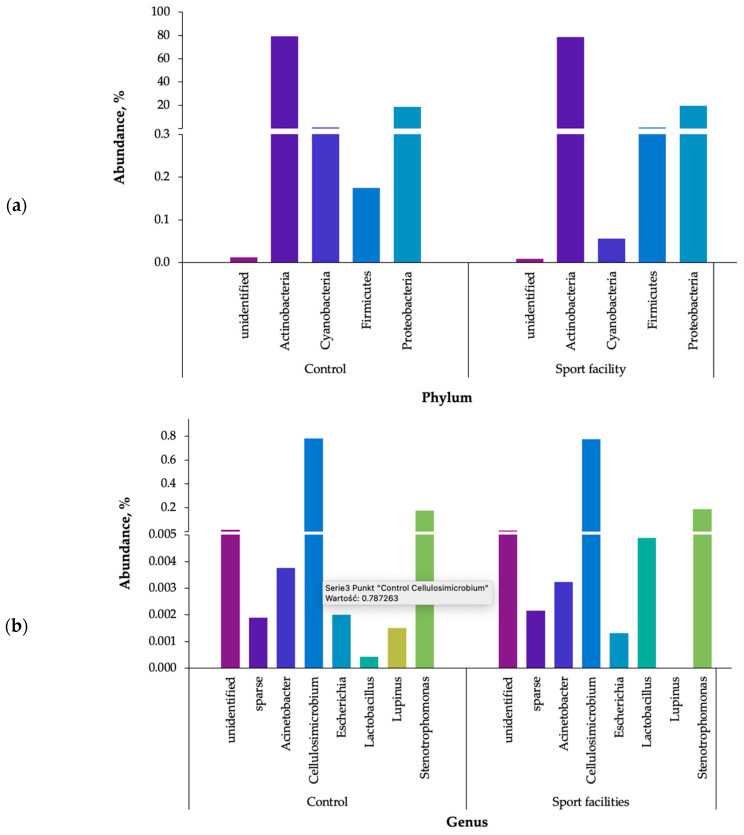
Phylogenetic distribution of bacteria sequences in the bioaerosols samples; (**a**) assigned to the phyla, (**b**) assigned to the genera; unidentified—unidentified sequences; sparse—reads assigned to low abundant phyla (less than 0.1% of all classified reads).

The results of identification from the high-throughput DNA sequencing of the atmospheric bioaerosol and internal air in the climbing wall hall of analysed locations are presented in Figure 6a (the phyla level) and Figure 6b (the genera level). In total, 43 genera of bacteria representing 5 phyla were detected in the analysed bioaerosol samples (Table S4). Most of the identified genera shared a small number of classified reads. The dominated phyla belonged to Actinobacteria (control air: 79.5%; sport facilities air: 78.8%) and proteobacteria (19.02% and 19.86%, respectively). Moreover, cyanobacteria (mainly in indoor air) and Firmicutes were detected with an abundance of 0.1–1.3% (Figure 6a).

The most abundant bacteria identified in bioaerososl samples belonged to the genera of *Cellulosimicrobium* (control air: 78.7%; climbing wall hall: 77.9%), *Stenotrophomonas* (18.2% and 19.3%, respectively), *Acinetobacter* (0.4% and 0.3%, respectively), and *Escherichia* (0.2% and 0.1%, respectively). *Lactobacillus* was found with high abundance in sports facilities (0.5%) but constituted only 0.04% of the control air. Other identified genera were present with less than 0.1% abundance (Table S4).

According to earlier studies, conducted with classical, culture-based methods, *Bacillus, Corynebacterium, Kocuria, Micrococcus, Pseudomonas*, and *Staphylococcus* are characteristic bacteria in the context of sports objects [16,54,56]. Identification based on gene sequences of 16S rRNA of isolates from gyms determined the phylogenetic affiliation of the detected bacteria to the following genera: *Bacillus, Brachybacterium, Geobacillus, Microbacterium, Micrococcus*, and *Staphylococcus* [64]. The obtained results differ from previous studies and indicate the presence of previously unidentified bacteria in the environment of sports facilities.

*Cellulosimicrobium* sp. are widely distributed in the soil and water; however, they have also been found to be part of the salivary microbiome [65,66,67]. This genus can cause infections in humans (Rowlinson et al., 2006). Similarly, the *Stenotrophomonas* bacteria found in the present work may be pathogenic (*S. maltophilia*), as they are commonly found in humid environments (soil, plants, fruit, sewage, and water). In the case of pathogenic strains, they are often associated with the hospital environment (Hamdi et al., 2020). Bacteria belonging to the genera *Acinetobacter* are widespread in the environment (soil and water). They are also part of the physiological microbiota of the skin and mucosa of the human nasopharynx [68]. Acinetobacter is often antibiotic-resistant pathogens responsible for human infections in the blood, urinary tract, and lungs (pneumonia).

*Escherichia* sp. have already been listed in the air of sports facilities. Mansoorian et al. (2015) identified it in indoor swimming pools. In the present research, bacteria from the *Enterobacteriaceae* family, including *E. coli*, were also detected in low concentrations using the culture method. It is noteworthy that pathogenic *Escherichia* strains are classified as harmful biological agents (group 2) according to Directive EU 2019/1833 [69].

*Lactobacillus* bacteria dominated the air near the climbing wall compared to the atmospheric air. *Lactobacillus* are found in different body sites, including the gastrointestinal tract, urinary tract, skin, and in breast milk [70]. Most probably, the presence of these bacteria is related to the activity of people in the examined room and the emission of their natural microbiota to the bioaerosol.

The literature pays attention to the presence of numerous pathogenic bacteria of *Staphylococcus aureus* in sport facilities [16]. In our research, we detected a large number of mannitol-positive staphylococci and *Staphylococcus aureus* bacteria using the culture method. Metagenomic analysis confirmed the presence of this type of bacteria, but their share in the total number was very low (less than 0.03% of all classified reads).

**Figure 7:**
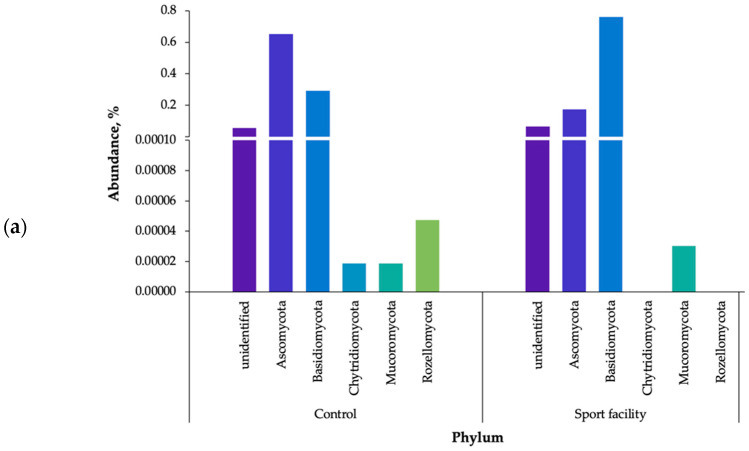

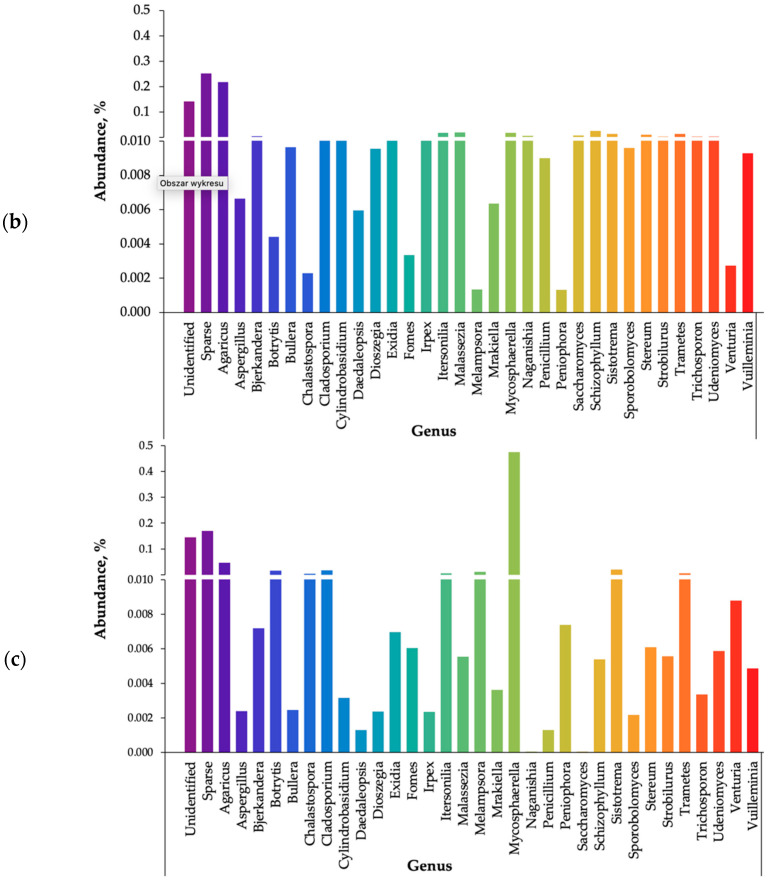
Phylogenetic distribution of fungi sequences in the settled dust sample; (**a**) assigned to the phyla, (**b**) assigned to the genera in sport facility; (**c**) assigned to the genera in control air; unidentified—unidentified sequences; sparse—reads assigned to low abundant phyla (less than 0.5% of all classified reads).

Metagenome analysis of bioaerosol samples showed greater biodiversity in terms of fungi than bacteria in both air samples. The ITS-based analysis revealed that, in total, 302 genera of fungi representing 7 phyla were detected in the analysed bioaerosol samples. The most abundant phyla belonged to *Ascomycota* (control air: 65.1%, climbing wall hall: 17.4%) and *Basidiomycota* (29.3 and 76.0%, respectively) (Figure 7a).

The most abundant fungi identified in both air samples belonged to the genera of *Mycosphaerella* (control air: 48.4%, climbing wall hall: 3.0), *Botrytis* (2.6% and 0.4%, respectively), *Chalastospora* (1.5% and 0.2%, respectively), *Cladosporium* (2.7% and 1.1%, respectively), and *Itersonilia* (1.6% and 3.0%, respectively). Additionally, *Melampsora* (2.2%) was found in control bioaerosol, while bioaerosol *Malassezia* (3.1%), *Naganishia* (1.7%), *Saccharomyces* (2%), *Sporobolomyces* (1.0%), *Trichosporon* (1.5%), and *Udeniomyces* (1.6%) were detected in climbing wall hall (Table S5, Figure 7b).

Fungi identification in sports facilities has not been the subject of many studies. The yeast of genera *Candida* sp., *Cryptococcus uniguttulattus*, *Rhodotorula*, and *Trichosporon mucoides* and the moulds *Acremonium*, *Alternaria*, *Aureobasidium*, *Cladosporium*, *Chrysonilia*, *Fusarium*, *Mucor*, *Penicillium*, and *Phoma* and were found in earlier studies conducted in these kinds of object worldwide [16,71].

The results obtained in the present study differ from the data in the literature. We found species not previously described for sports facilities, namely *Malassezia, Naganishia, Saccharomyces, Sporobolomyces,* and *Udeniomyces*. These fungi are common genera and are likely to come from the surrounding environment, mainly from atmospheric air.

*Sporobolomyces* are ubiquitous fungi commonly isolated from environmental sources, such as the air, tree leaves, soil, rotting fruit, and other plant materials. *Sporobolomyces* may cause infections, particularly in immunosuppressed patients [72].

*Saccharomyces* is one of the types of yeast extensively found in naturally occurring ecosystems such as the air, soil, and food products. It is mainly implicated in the fermentative spoilage of high-sugar foods and beverages [73].

Yeast of the genus *Malassezia* are a species specific to the human skin and can be isolated from the surface of the whole body. They are associated with the occurrence of many dermatological diseases, both as an etiological factor in fungal infection (tinea versicolor, folliculitis) and as a pathogen that intensifies skin lesions [74].

Among the fungi identified with the highest number of readings, *Malassezia* yeasts are of human origin, with the remaining genera most likely being related to atmospheric air. In turn, *Naganishia* (earlier *Cryptococcus)* includes 15 species of asexual yeast reported from different ecosystems including water, soil, glaciers, fermented cereals, air, purification tanks, and diseased human fingernails [75].

It is worth noting that plant pollen (*Lupinus*) and basidiomycete-producing fruiting body (basidiocarp) were also identified, specifically *Agaricus, Cylindrobasidium, Exidia, Strobilurus, Sistotrema Schizophyllum Stereum, Strobilurus, Trametes* (Table S5, Figure 7b,c).

## 4. Conclusions

The analysis of indoor air in the sports activity rooms (the climbing wall, swimming pool, changing room at the swimming pool, basketball/volleyball court, badminton court) at the Sports Centre of the Lodz University of Technology (Zatoka Sportu) showed that selected parameters, namely microclimatic (temperature and humidity), particulate (PM_10_, PM_2.5_, and PM_1_), and microbiological (the total number of bacteria, total number of fungi, and *Staphylococcus aureus*) may be indicators of the air quality of sports facilities. It was found that the limit ranges of the above parameters were exceeded, mainly at the climbing wall and in the changing room at the swimming pool, where the worst indoor air quality among the rooms surveyed was found.

The air temperature in the examined sports premises was within the upper ranges of thermal comfort (21–31 °C), while the air humidity in the climbing wall hall and on the playing fields was too low (RH < 40%). With high physical activity, such parameters can reduce the thermal comfort of people practising sports. Moreover, a high concentration of dust in the indoor air was observed. The measured air dustiness of all tested fractions in the tested sports premises exceeded the WHO guidelines; the highest exceedances of standards occurred for the PM_2.5_ fraction (five-fold) and for the PM_10_ fraction (two-fold). Particularly hazardous to health is respirable fraction particulate matter (PM_4_, PM_2.5_, and PM_1_), for which high air concentrations were also detected. Attempts should be made to reduce dust by using new filters and increasing the frequency of room cleaning.

The maximum reduction in oxygen concentration in the air of the examined sports rooms was 1% from the correct level of 21%; these are not differences perceptible to humans. Concentrations of chemical parameters (ozone, hydrogen sulphide, and sulphur dioxide) in the air did exceed WHO limits at any point. Elevated instantaneous benzopyrene concentrations in the outdoor air and in the climbing wall hall were associated with incomplete combustion processes that do not occur in the facility, so infiltration from the outside is the likely cause. Getting rid of the source of the contamination can be problematic, and for this reason it is necessary to monitor the concentration of benzopyrene and find the source of the contamination. A possible solution is to temporarily create an overpressure in the premises so that when this toxic hydrocarbon is at its highest concentrations of it does not penetrate into the building.

Exceeded limits of contamination by bacteria and fungi in the air and elevated levels of staphylococci in the air of the surveyed premises, including *Staphylococcus aureus* species, were found. This is related to the high number of people present/playing sports. Certain species of staphylococci may be pathogenic, so when there are elevated concentrations of these bacteria in the air, more frequent cleaning and the periodic disinfection of the premises should be implemented. An increased number of fungi, including xerophilic fungi, was observed in the air, which was related to the high dust content and low humidity.

The study proved that as the number of users of the facility in the afternoon and their sporting activities increased, the concentration of dust (several times) and microorganisms in the air also increased (by one–two orders of magnitude). Analysis of the microbiological biodiversity of the air in the sports premises using molecular biology methods revealed numerous species of bacteria and fungi from both humans and the atmospheric environment.

This comprehensive study has not only indicated which parameters should be monitored to assess air quality but has also provided guidelines on how to improve the comfort of those who practice sports and work in sports premises.

Effective air quality management in sports facilities includes installing appropriate monitoring systems to control the amount of dust, humidity, and air temperature. Periodically, the microbiological air contamination should be determined, particularly in places where a large number of people exercise. Software tools allowing for the simulation and prediction of the examined parameters in changing conditions can be applied. Effective air quality management comprises the identification of the most effective methods of removing air pollutants and regulating air parameters in sports facilities. These include the installation of an individually designed HVAC system, taking into account the specificity of a place with increased air exchange. Moreover, the installation of local ventilation in areas with high dust or curtains and walls, allowing for the mechanical separation of places with different air qualities. In the case of removing microbial contamination, more frequent cleaning and disinfection should be considered, especially during the increased usage of sports facilities. Future studies on the quality of the indoor environment conducted in sports facilities should include a post-occupancy evaluation (POE) with particular emphasis on real performances as experienced by the users over a long period of time [76]. Another need is to develop a system of multifunctional sensors to measure air quality in a continuous system and warn users against exceeding the permissible limits for individual parameters.

## Figures and Tables

**Table 1:** Characteristics of the analysed sports facilities.

Place	Location	Cubature, m^3^	Number of People	Analysed Surfaces for Microbiological Study
A	Climbing wall	5200	Morning: 2–6 Afternoon: 10–30	(1) holder on the wall; (2) wall surface; (3) table; (4) locker for handy things; (5) reception desk
B	Swimming pool	45,400	Morning: 10–20 Afternoon: 30–50	(6) starting post no. 1; (7) starting post no. 4; (8) starting post no. 8; (9) seating area (between lanes 7 and 8)
C	Changing room at the swimming pool	1600	Morning: 2–10 Afternoon: 20–40	(10) place to sit under the locker no. 110; (11) inside locker no. 107; (12) bench in the lockable changing room; (13) mirror on the left side of the room; (14) paper towel dispenser; (15) external door of cabinet no. 65;
D	Basketball/volleyball court	13,800	Morning: 2–10 Afternoon: 20–30	(16) cover for the basketball basket structure; (17) table by the stands; (18) seat in the stands
E	Badminton court	2400	Morning: 0–2 Afternoon: 10–20	(19) seat for the followers; (20) court floor
F	Atmospheric air in front of the building	nt*		

nt* not tested—control (external background) samples.

## Data Availability

The data presented in this study are available on request from the corresponding author.

## Supplementary Materials

The following supporting information can be downloaded at: https://www.mdpi.com/article/10.3390/ijerph20021551/s1, Table S1. Microclimate parameters in the analysed locations; Table S2. Oxygen and chemical contaminants concentration in the analysed locations; Table S3. Number of microorganisms in the analysed sport facilities; Table S4. Taxonomy of bacteria detected in the analysed facilities; Table S5. Taxonomy of fungi detected in the analysed facilities; Figure S1. Microbiological contamination of surfaces.
