# Supplementary material for: Assessment of Dust, Chemical, Microbiological Pollutions and Microclimatic Parameters of Indoor Air in Sports Facilities
Source: Int J Environ Res Public Health. 2023 Jan 14;20(2):1551. doi: 10.3390/ijerph20021551 (PMC9865041; doi:10.3390/ijerph20021551)
Supplement: Supplementary file 1 [file ijerph-20-01551-s001.zip › Figure S1.pdf]

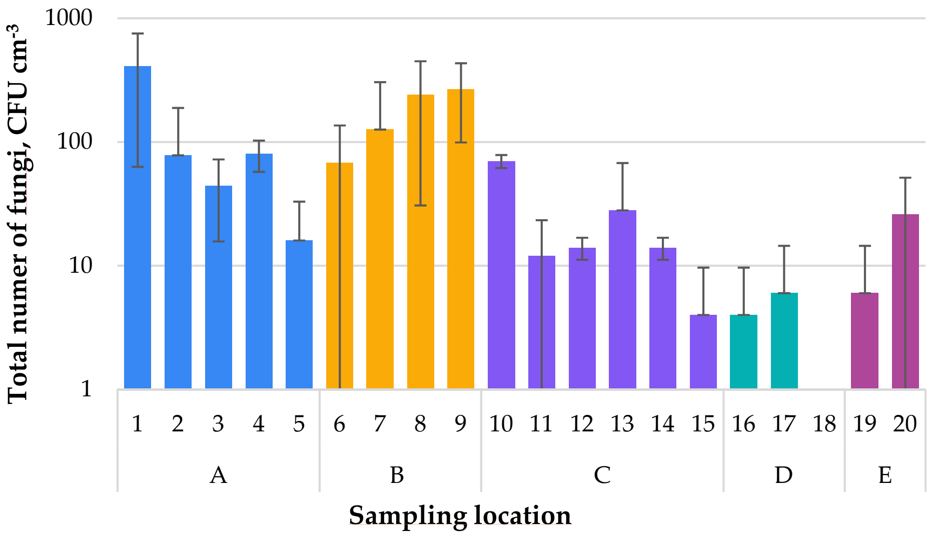

(a)

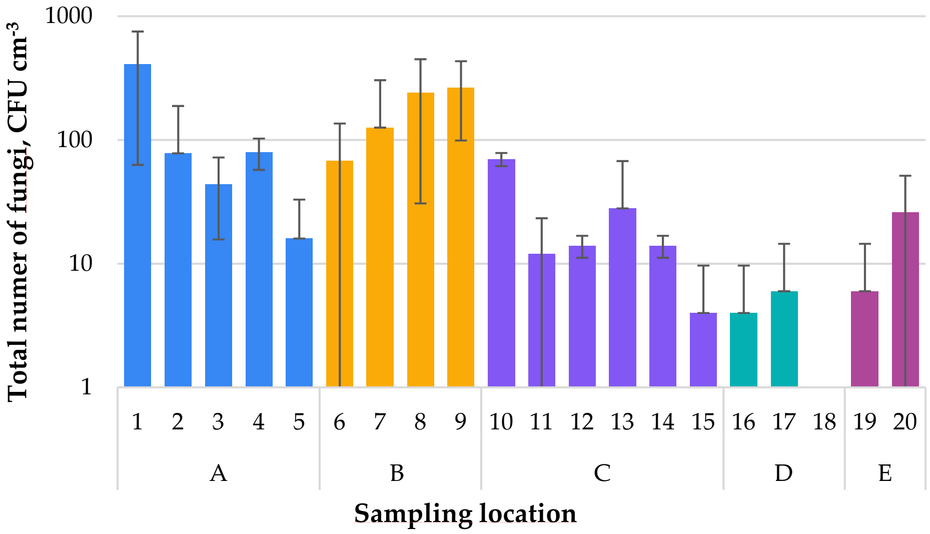

(b)

Figure S1. Microbiological contamination of surfaces: a). Bacteria; b). Fungi (1) holder on the wall; (2) wall surface; (3)table; (4)cabinet for handy things; (5)reception desk; (6) starting post no. 1; (7) starting post no. 4; (8)starting post no. 8; (9)seating area (between lanes 7 and 8); (10- a place to sit under the cupboard no. 110; (11) inside the cabinet no.107; (12) a bench in a lockable changing room; (13) mirror on the left side of the room; (14) paper towel dispenser; (15)external door of the cabinet no. 65; (16) cover for the basketball basket structure; (17) a table by the stands; (18) the seat in the stands; (19) a seat for the followers; (20)-court floor
