# Supplementary material for: Assessment of Dust, Chemical, Microbiological Pollutions and Microclimatic Parameters of Indoor Air in Sports Facilities
Source: Int J Environ Res Public Health. 2023 Jan 14;20(2):1551. doi: 10.3390/ijerph20021551 (PMC9865041; doi:10.3390/ijerph20021551)
Supplement: Supplementary file 1 [file ijerph-20-01551-s001.zip › Table S1.pdf]

Table S1. Microclimate parameters in tested locations

Sampling location	Time	Date	Temperature (^oC)				Relative humidity (%)				Airflow velocity (m/s)			
			Medium	Minimum	Maksimum	Standard deviation	Medium	Minimum	Maksimum	Standard deviation	Medium	Minimum	Maksimum	Standard deviation
A	M	05.05.2022	**21.5**	21.0	21.7	0.3	**35.4**	33.8	37.0	1.3	**0.2**	0.1	0.3	0.1
A	A	05.05.2022	**23.3**	23.2	23.3	0.0	**32.7**	32.4	33.1	0.3	**0.2**	0.1	0.2	0.0
A	M	12.05.2022	**22.9**	22.7	23.0	0.1	**44.2**	43.7	44.7	0.4	**0.1**	0.0	0.2	0.0
A	A	12.05.2022	**23.9**	23.6	24.2	0.2	**35.5**	35.1	35.7	0.2	**0.0**	0.0	0.1	0.0
A	M	19.05.2022	**22.6**	22.4	22.8	0.1	**23.8**	23.5	24.0	0.2	**0.1**	0.0	0.2	0.0
A	A	19.05.2022	**24.1**	24.0	24.1	0.1	**25.0**	24.9	25.1	0.1	**0.1**	0.0	0.1	0.0
B	M	05.05.2022	**28.5**	27.5	29.2	0.7	**50.6**	45.8	53.6	2.9	**0.2**	0.1	0.2	0.1
B	A	05.05.2022	**28.6**	27.8	29.4	0.7	**64.8**	62.4	67.8	1.8	**0.1**	0.1	0.3	0.1
B	M	12.05.2022	**28.8**	28.3	29.2	0.3	**61.4**	58.1	65.1	2.9	**0.0**	0.0	0.0	0.0
B	A	12.05.2022	**28.9**	28.7	29.1	0.2	**55.7**	47.0	64.9	9.4	**0.1**	0.0	0.1	0.0
B	M	19.05.2022	**29.1**	28.5	29.5	0.4	**50.6**	48.3	54.8	2.6	**0.1**	0.0	0.1	0.0
B	A	19.05.2022	**28.8**	28.5	29.2	0.2	**55.3**	48.1	62.4	7.3	**0.1**	0.1	0.2	0.0
C	M	05.05.2022	**25.9**	25.8	26.0	0.1	**30.8**	30.4	31.2	0.4	**0.1**	0.0	0.1	0.0
C	A	05.05.2022	**26.7**	26.5	26.8	0.2	**32.0**	31.9	32.1	0.1	**0.2**	0.2	0.2	0.0
C	M	12.05.2022	**25.8**	25.6	25.9	0.2	**31.2**	30.5	31.6	0.6	**0.1**	0.0	0.1	0.0
C	A	12.05.2022	**27.0**	27.0	27.1	0.1	**33.9**	33.5	34.5	0.5	**0.2**	0.1	0.2	0.0
C	M	19.05.2022	**25.6**	25.4	25.8	0.2	**33.1**	32.4	33.5	0.6	**0.1**	0.0	0.2	0.1
C	A	19.05.2022	**27.1**	27.1	27.1	0.0	**35.4**	35.3	35.5	0.1	**0.2**	0.2	0.2	0.0
D	M	05.05.2022	**22.2**	22.1	22.3	0.1	**33.8**	33.7	33.9	0.1	**0.1**	0.1	0.1	0.0
D	A	05.05.2022	**23.7**	23.6	23.7	0.1	**34.7**	34.5	34.8	0.2	**0.2**	0.1	0.3	0.1
D	M	12.05.2022	**23.1**	23.0	23.2	0.1	**42.5**	42.3	42.7	0.2	**0.0**	0.0	0.0	0.0
D	A	12.05.2022	**24.0**	23.8	24.3	0.3	**32.4**	30.8	33.5	1.4	**0.2**	0.2	0.2	0.0
D	M	19.05.2022	**22.6**	22.5	22.6	0.1	**23.6**	23.5	23.7	0.1	**0.1**	0.1	0.1	0.0
D	A	19.05.2022	**24.0**	24.0	24.1	0.1	**24.6**	24.6	24.7	0.1	**0.1**	0.1	0.1	0.0
E	M	05.05.2022	**22.3**	22.2	22.4	0.1	**27.4**	26.6	27.9	0.7	**0.1**	0.1	0.1	0.0
E	A	05.05.2022	**22.9**	22.6	23.1	0.3	**31.0**	30.3	32.2	1.0	**0.1**	0.1	0.1	0.0
E	M	12.05.2022	**23.6**	23.4	23.8	0.2	**40.8**	40.4	41.3	0.5	**0.0**	0.0	0.0	0.0
E	A	12.05.2022	**24.7**	24.6	24.7	0.1	**36.3**	36.0	36.5	0.3	**0.0**	0.0	0.0	0.0
E	M	19.05.2022	**23.5**	23.5	23.6	0.1	**23.1**	22.9	23.3	0.2	**0.1**	0.0	0.1	0.0
E	A	19.05.2022	**24.7**	24.6	24.8	0.1	**27.4**	27.3	27.5	0.1	**0.1**	0.0	0.1	0.0
F	M	05.05.2022	**11.9**	11.7	12.2	0.3	**54.8**	52.7	56.1	1.8	**2.0**	1.9	2.1	0.1
F	M	12.05.2022	**20.0**	19.9	20.0	0.1	**49.7**	49.2	50.0	0.4	**3.7**	2.0	5.0	1.5
F	M	19.05.2022	**14.6**	14.5	14.7	0.1	**32.1**	31.5	32.5	0.5	**4.4**	3.5	5.6	1.1

Sampling location: A - climbing wall; B - swimming pool; C - changing room at the swimming pool; D - basketball / volleyball court; E - badminton court; F - atmospheric air in front of Zatoka Sportu building; Time: M - morning; A - afternoon
