# Supplementary material for: Assessment of Dust, Chemical, Microbiological Pollutions and Microclimatic Parameters of Indoor Air in Sports Facilities
Source: Int J Environ Res Public Health. 2023 Jan 14;20(2):1551. doi: 10.3390/ijerph20021551 (PMC9865041; doi:10.3390/ijerph20021551)
Supplement: Supplementary file 1 [file ijerph-20-01551-s001.zip › Table S2.pdf]

Table S2. Oxygen and chemical contaminants concentration at the tested locations

Sampling location	Sampling date	Sampling time	O_2 [%]		H_2S [ppm]		SO_2 [ppm]		Odorous compounds [ppm]		O_3 [ppm]		VOC* [ppm]	
			M	SD	M	SD	M	SD	M	SD	M	SD	M	SD
**A**	05.05.2022	M	20.3864	0.1218	0.0764	0.0050	0.0450	0.0565	0.0000	0.0000	0.0000	0.0000	0.0092	0.0075
		A	20.8608	0.1332	0.1063	0.1332	0.1196	0.1437	184.9806	69.9245	0.0000	0.0000	0.0014	0.0018
	12.05.2022	M	20.3239	0.0749	0.0868	0.0166	0.0012	0.0031	232.2999	49.1481	0.1088	0.0499	0.0355	0.0009
		A	21.2005	0.1345	0.1278	0.0321	0.1270	0.1471	258.1103	0.0005	0.0408	0.0126	0.0003	0.0002
	19.05.2022	M	20.1202	0.0765	0.0592	0.0034	0.0000	0.0001	258.1097	0.0004	0.0664	0.0392	0.0003	0.0004
		A	20.5863	0.0095	0.1208	0.0045	0.0033	0.0020	0.0000	0.0000	0.0406	0.0274	0.0055	0.0010
**D**	05.05.2022	M							nt					
		A												
	12.05.2022	M	20.2701	0.0133	0.1317	0.0061	0.0037	0.0004	197.8861	81.6219	0.0634	0.0269	0.0342	0.0001
		A												
	19.05.2022	M	19.8973	0.0159	0.0819	0.0065	0.0006	0.0008	258.1100	0.0008	0.0626	0.0500	0.0004	0.0004
		A	20.5480	0.0302	0.1002	0.0140	0.0028	0.0025	0.7087	0.0037	0.0429	0.0238	0.0015	0.0008
**E**	05.05.2022	M												
		A							nt					
	12.05.2022	M												
		A	20.6144	0.0188	0.1053	0.0185	0.0026	0.0018	0.0000	0.0000	0.0232	0.0156	0.0032	0.0002
	19.05.2022	M	19.6014	0.0905	0.1831	0.0102	0.0015	0.0006	0.0000	0.0000	0.0427	0.0335	0.0006	0.0004
		A	20.9196	0.1760	0.0815	0.0129	0.1310	0.0844	258.1102	0.0266	0.0485	0.0266	0.0003	0.0003
**F**	05.05.2022	M	20.7902	0.0736	0.1162	0.0155	0.5076	0.0616	258.1109	0.0007	0.0000	0.0000	0.0007	0.0009
		A							nt					
	12.05.2022	M	20.7177	0.1280	0.2419	0.0842	0.5266	0.1154	258.1106	0.0007	0.0344	0.0090	0.0003	0.0001
		A							nt					
	19.05.2022	M	20.5941	0.0680	0.0636	0.0104	0.3363	124.6789	77.4329	124.6789	0.0787	0.0239	0.0015	0.0013
		A							nt					

Sampling location: A - climbing wall; D - basketball / volleyball court; E - badminton court; F - atmospheric air in front of Zatoka Sportu building; Time: M - morning; A – afternoon; M – mean; SD- standard deviation; nt – not tested; VOC* - only benzo-α-pyrene was detected
