# Supplementary material for: Assessment of Dust, Chemical, Microbiological Pollutions and Microclimatic Parameters of Indoor Air in Sports Facilities
Source: Int J Environ Res Public Health. 2023 Jan 14;20(2):1551. doi: 10.3390/ijerph20021551 (PMC9865041; doi:10.3390/ijerph20021551)
Supplement: Supplementary file 1 [file ijerph-20-01551-s001.zip › Table S3.pdf]

Table S3. Number of microorganisms in tested sport facilities

Locat ion	Date	Time	Bacteria		Mannitol-positive Staphylococcus		*Staphylococcus aureus*		Haemolytic Staphylococcus		*Enterobacteriaceae*		*Pseudomonas fluorescens*		Actinomycetes		Fungi		Xerophilic fungi	
			M	SD	M	SD	M	SD	M	SD	M	SD	M	SD	M	SD	M	SD	M	SD
	05.05.	M	$2.23x10^{2}$	$5.56x10^{1}$	$1.50x10^{1}$	$5.77x10^{0}$	$3.50x10^{1}$	$1.73x10^{1}$	$5.00x10^{0}$	$5.77x10^{0}$	$0.00x10^{0}$	$0.00x10^{0}$	$2.50x10^{0}$	$5.00x10^{0}$	$0.00x10^{0}$	$0.00x10^{0}$	$1.00x10^{2}$	$0.00x10^{0}$	$5.00x10^{0}$	$1.00x10^{1}$
	2022	A	$2.42x10^{3}$	$1.76x10^{3}$	$2.08x10^{2}$	$2.22x10^{1}$	$5.25x10^{2}$	$1.45x10^{2}$	$7.50x10^{1}$	$1.29x10^{1}$	$1.25x10^{1}$	$5.00x10^{0}$	$0.00x10^{0}$	$0.00x10^{0}$	$0.00x10^{0}$	$0.00x10^{0}$	$5.00x10^{1}$	$8.16x10^{0}$	$1.25x10^{1}$	$1.89x10^{1}$
**A**	12.05.	M	$1.80x10^{2}$	$9.49x10^{1}$	$2.25x10^{1}$	$1.26x10^{1}$	$2.00x10^{1}$	$1.15x10^{1}$	$7.50x10^{0}$	$9.57x10^{0}$	$0.00x10^{0}$	$0.00x10^{0}$	$0.00x10^{0}$	$0.00x10^{0}$	$2.50x10^{0}$	$5.00x10^{0}$	$1.00x10^{1}$	$1.15x10^{1}$	$1.25x10^{1}$	$5.00x10^{0}$
	2022	A	$3.81x10^{3}$	$2.06x10^{3}$	$9.75x10^{2}$	$4.88x10^{2}$	$6.40x10^{2}$	$3.07x10^{2}$	$2.50x10^{1}$	$5.77x10^{0}$	$1.50x10^{1}$	$5.77x10^{0}$	$0.00x10^{0}$	$0.00x10^{0}$	$2.00x10^{1}$	$8.16x10^{0}$	$7.50x10^{1}$	$2.38x10^{1}$	$7.00x10^{1}$	$3.56x10^{1}$
	19.05.	M	$2.63x10^{2}$	$1.13x10^{2}$	$6.00x10^{1}$	$5.35x10^{1}$	$3.75x10^{1}$	$4.35x10^{1}$	$1.50x10^{1}$	$1.29x10^{1}$	$2.50x10^{0}$	$5.00x10^{0}$	$0.00x10^{0}$	$0.00x10^{0}$	$2.50x10^{0}$	$5.00x10^{0}$	$1.25x10^{1}$	$9.57x10^{0}$	$1.25x10^{1}$	$1.50x10^{1}$
	2022	A	$1.22x10^{3}$	$2.41x10^{2}$	$1.48x10^{2}$	$3.59x10^{1}$	$3.78x10^{2}$	$3.77x10^{1}$	$3.00x10^{1}$	$8.16x10^{0}$	$7.50x10^{0}$	$9.57x10^{0}$	$0.00x10^{0}$	$0.00x10^{0}$	$5.00x10^{0}$	$5.77x10^{0}$	$1.75x10^{1}$	$1.50x10^{1}$	$2.75x10^{1}$	$2.36x10^{1}$
	05.05.	M	$5.13x10^{1}$	$2.02x10^{1}$	$2.50x10^{1}$	$1.29x10^{1}$	$2.00x10^{1}$	$8.16x10^{0}$	$0.00x10^{0}$	$0.00x10^{0}$	$0.00x10^{0}$	$0.00x10^{0}$	$5.00x10^{0}$	$1.00x10^{1}$	$5.00x10^{0}$	$5.77x10^{0}$	$1.00x10^{1}$	$8.16x10^{0}$	$2.50x10^{0}$	$5.00x10^{0}$
	2022	A	$5.33x10^{2}$	$5.31x10^{2}$	$5.75x10^{1}$	$2.75x10^{1}$	$6.75x10^{1}$	$6.13x10^{1}$	$1.50x10^{1}$	$5.77x10^{0}$	$2.50x10^{0}$	$5.00x10^{0}$	$2.50x10^{0}$	$5.00x10^{0}$	$0.00x10^{0}$	$0.00x10^{0}$	$2.50x10^{1}$	$1.73x10^{1}$	$1.75x10^{1}$	$2.06x10^{1}$
**B**	12.05.	M	$2.03x10^{2}$	$1.18x10^{2}$	$2.50x10^{1}$	$1.91x10^{1}$	$2.25x10^{1}$	$9.57x10^{0}$	$1.00x10^{1}$	$8.16x10^{0}$	$2.50x10^{0}$	$5.00x10^{0}$	$5.00x10^{0}$	$1.00x10^{1}$	$2.50x10^{0}$	$5.00x10^{0}$	$3.50x10^{1}$	$1.00x10^{1}$	$2.75x10^{1}$	$2.06x10^{1}$
	2022	A	$3.70x10^{2}$	$1.36x10^{2}$	$7.00x10^{1}$	$5.83x10^{1}$	$3.50x10^{1}$	$2.38x10^{1}$	$2.50x10^{1}$	$5.77x10^{0}$	$2.50x10^{0}$	$5.00x10^{0}$	$0.00x10^{0}$	$0.00x10^{0}$	$0.00x10^{0}$	$0.00x10^{0}$	$4.25x10^{1}$	$3.20x10^{1}$	$1.00x10^{1}$	$8.16x10^{0}$
	19.05.	M	$2.95x10^{2}$	$3.79x10^{1}$	$1.45x10^{2}$	$1.11x10^{2}$	$6.75x10^{1}$	$2.75x10^{1}$	$3.25x10^{1}$	$9.57x10^{0}$	$0.00x10^{0}$	$0.00x10^{0}$	$2.50x10^{0}$	$5.00x10^{0}$	$5.00x10^{0}$	$5.77x10^{0}$	$1.75x10^{1}$	$9.57x10^{0}$	$1.75x10^{1}$	$9.57x10^{0}$
	2022	A	$8.28x10^{2}$	$4.20x10^{2}$	$1.35x10^{2}$	$2.89x10^{1}$	$7.50x10^{1}$	$4.04x10^{1}$	$2.50x10^{1}$	$5.77x10^{0}$	$0.00x10^{0}$	$0.00x10^{0}$	$0.00x10^{0}$	$0.00x10^{0}$	$5.00x10^{0}$	$5.77x10^{0}$	$3.25x10^{1}$	$1.26x10^{1}$	$4.50x10^{1}$	$1.91x10^{1}$
	05.05.	M	$1.87x10^{3}$	$1.27x10^{2}$	$2.50x10^{1}$	$7.07x10^{0}$	$7.50x10^{1}$	$2.12x10^{1}$	$0.00x10^{0}$	$0.00x10^{0}$	$0.00x10^{0}$	$0.00x10^{0}$	$0.00x10^{0}$	$0.00x10^{0}$	$1.00x10^{1}$	$0.00x10^{0}$	$1.20x10^{2}$	$1.41x10^{1}$	$2.00x10^{1}$	$1.41x10^{1}$
	2022	A	$1.26x10^{4}$	$7.21x10^{2}$	$3.55x10^{2}$	$1.91x10^{2}$	$2.23x10^{3}$	$5.02x10^{2}$	$3.00x10^{1}$	$1.41x10^{1}$	$5.00x10^{1}$	$1.41x10^{1}$	$5.00x10^{0}$	$7.07x10^{0}$	$0.00x10^{0}$	$0.00x10^{0}$	$1.20x10^{2}$	$0.00x10^{0}$	$3.00x10^{1}$	$0.00x10^{0}$
**C**	12.05.	M	$3.15x10^{2}$	$2.12x10^{1}$	$3.50x10^{1}$	$2.12x10^{1}$	$3.50x10^{1}$	$7.07x10^{0}$	$5.00x10^{0}$	$7.07x10^{0}$	$0.00x10^{0}$	$0.00x10^{0}$	$0.00x10^{0}$	$0.00x10^{0}$	$0.00x10^{0}$	$0.00x10^{0}$	$3.00x10^{1}$	$0.00x10^{0}$	$5.50x10^{1}$	$4.95x10^{1}$
	2022	A	$2.04x10^{4}$	$8.15x10^{3}$	$1.00x10^{3}$	$4.38x10^{2}$	$2.35x10^{3}$	$5.09x10^{2}$	$2.50x10^{1}$	$7.07x10^{0}$	$5.00x10^{0}$	$7.07x10^{0}$	$2.00x10^{1}$	$0.00x10^{0}$	$4.00x10^{1}$	$0.00x10^{0}$	$1.70x10^{2}$	$0.00x10^{0}$	$3.75x10^{2}$	$7.78x10^{1}$
	19.05.	M	$7.86x10^{3}$	$2.19x10^{2}$	$3.05x10^{2}$	$1.06x10^{2}$	$1.70x10^{2}$	$2.83x10^{1}$	$2.50x10^{1}$	$7.07x10^{0}$	$1.50x10^{1}$	$7.07x10^{0}$	$5.00x10^{0}$	$7.07x10^{0}$	$1.00x10^{1}$	$0.00x10^{0}$	$1.50x10^{2}$	$7.07x10^{1}$	$1.50x10^{2}$	$7.07x10^{1}$
	2022	A	$1.36x10^{4}$	$7.07x10^{2}$	$8.95x10^{2}$	$1.20x10^{2}$	$6.10x10^{2}$	$2.83x10^{1}$	$2.50x10^{1}$	$7.07x10^{0}$	$3.00x10^{1}$	$0.00x10^{0}$	$5.00x10^{0}$	$7.07x10^{0}$	$5.00x10^{0}$	$7.07x10^{0}$	$2.70x10^{2}$	$2.83x10^{1}$	$2.00x10^{2}$	$7.07x10^{1}$
	05.05.	M	$2.90x10^{2}$	$4.24x10^{1}$	$1.50x10^{1}$	$7.07x10^{0}$	$2.50x10^{1}$	$7.07x10^{0}$	$0.00x10^{0}$	$0.00x10^{0}$	$0.00x10^{0}$	$0.00x10^{0}$	$0.00x10^{0}$	$0.00x10^{0}$	$0.00x10^{0}$	$0.00x10^{0}$	$2.50x10^{1}$	$7.07x10^{0}$	$1.00x10^{1}$	$0.00x10^{0}$
	2022	A	$2.13x10^{3}$	$5.16x10^{2}$	$2.50x10^{2}$	$4.24x10^{1}$	$4.05x10^{2}$	$4.95x10^{1}$	$3.50x10^{1}$	$7.07x10^{0}$	$2.00x10^{1}$	$0.00x10^{0}$	$0.00x10^{0}$	$0.00x10^{0}$	$0.00x10^{0}$	$0.00x10^{0}$	$4.00x10^{1}$	$0.00x10^{0}$	$2.50x10^{1}$	$7.07x10^{0}$
**D**	12.05.	M	$4.80x10^{2}$	$2.69x10^{2}$	$2.00x10^{1}$	$1.41x10^{1}$	$2.50x10^{1}$	$7.07x10^{0}$	$0.00x10^{0}$	$0.00x10^{0}$	$0.00x10^{0}$	$0.00x10^{0}$	$0.00x10^{0}$	$0.00x10^{0}$	$0.00x10^{0}$	$0.00x10^{0}$	$4.50x10^{1}$	$7.07x10^{0}$	$4.50x10^{1}$	$7.07x10^{0}$
	2022	A	$1.37x10^{3}$	$2.19x10^{2}$	$2.60x10^{2}$	$1.13x10^{2}$	$2.45x10^{2}$	$1.06x10^{2}$	$3.50x10^{1}$	$7.07x10^{0}$	$3.00x10^{1}$	$1.41x10^{1}$	$5.00x10^{0}$	$7.07x10^{0}$	$5.00x10^{0}$	$7.07x10^{0}$	$2.50x10^{1}$	$7.07x10^{0}$	$2.50x10^{1}$	$7.07x10^{0}$
	19.05.	M	$1.65x10^{2}$	$2.12x10^{1}$	$3.50x10^{1}$	$7.07x10^{0}$	$3.50x10^{1}$	$7.07x10^{0}$	$1.00x10^{1}$	$0.00x10^{0}$	$0.00x10^{0}$	$0.00x10^{0}$	$0.00x10^{0}$	$0.00x10^{0}$	$0.00x10^{0}$	$0.00x10^{0}$	$2.50x10^{1}$	$2.12x10^{1}$	$2.50x10^{1}$	$2.12x10^{1}$
	2022	A	$1.16x10^{3}$	$1.20x10^{2}$	$1.50x10^{2}$	$2.83x10^{1}$	$2.75x10^{2}$	$3.54x10^{1}$	$1.00x10^{1}$	$1.41x10^{1}$	$0.00x10^{0}$	$0.00x10^{0}$	$0.00x10^{0}$	$0.00x10^{0}$	$0.00x10^{0}$	$0.00x1^{00}$	$2.50x10^{1}$	$7.07x10^{0}$	$3.00x10^{1}$	$1.41x10^{1}$
	05.05.	M	$3.25x10^{2}$	$9.19x10^{1}$	$1.50x10^{1}$	$7.07x10^{0}$	$3.50x10^{1}$	$7.07x10^{0}$	$1.00x10^{1}$	$0.00x10^{0}$	$0.00x10^{0}$	$0.00x10^{0}$	$0.00x10^{0}$	$0.00x10^{0}$	$5.00x10^{0}$	$7.07x10^{0}$	$2.50x10^{1}$	$2.12x10^{1}$	$1.00x10^{1}$	$0.00x10^{0}$
	2022	A	$7.52x10^{3}$	$7.86x10^{3}$	$1.70x10^{2}$	$2.83x10^{1}$	$3.60x10^{2}$	$1.84x10^{2}$	$3.50x10^{1}$	$7.07x10^{0}$	$5.00x10^{0}$	$7.07x10^{0}$	$0.00x10^{0}$	$0.00x10^{0}$	$0.00x10^{0}$	$0.00x10^{0}$	$2.50x10^{1}$	$7.07x10^{0}$	$3.50x10^{1}$	$7.07x10^{0}$
**E**	12.05.	M	$1.57x10^{3}$	$3.04x10^{2}$	$8.50x10^{1}$	$7.07x10^{0}$	$1.25x10^{2}$	$7.07x10^{0}$	$1.00x10^{1}$	$1.41x10^{1}$	$5.00x10^{0}$	$7.07x10^{0}$	$0.00x10^{0}$	$0.00x10^{0}$	$0.00x10^{0}$	$0.00x10^{0}$	$4.00x10^{1}$	$1.41x10^{1}$	$0.00x10^{0}$	$0.00x10^{0}$
	2022	A	$1.65x10^{3}$	$1.84x10^{2}$	$3.20x10^{2}$	$9.90x10^{1}$	$2.15x10^{2}$	$2.12x10^{1}$	$4.50x10^{1}$	$7.07x10^{0}$	$1.00x10^{1}$	$1.41x10^{1}$	$0.00x10^{0}$	$0.00x10^{0}$	$1.50x10^{1}$	$7.07x10^{0}$	$3.50x10^{1}$	$7.07x10^{0}$	$5.00x10^{0}$	$7.07x10^{0}$
	19.05.	M	$7.00x10^{1}$	$1.41x10^{1}$	$5.50x10^{1}$	$7.07x10^{0}$	$3.50x10^{1}$	$7.07x10^{0}$	$1.00x10^{1}$	$0.00x10^{0}$	$0.00x10^{0}$	$0.00x10^{0}$	$5.00x10^{0}$	$7.07x10^{0}$	$0.00x10^{0}$	$0.00x10^{0}$	$1.00x10^{1}$	$0.00x10^{0}$	$1.00x10^{1}$	$0.00x10^{0}$
	2022	A	$9.85x10^{2}$	$3.61x10^{2}$	$1.45x10^{2}$	$7.07x10^{0}$	$1.60x10^{2}$	$5.66x10^{1}$	$2.50x10^{1}$	$7.07x10^{0}$	$0.00x10^{0}$	$0.00x10^{0}$	$0.00x10^{0}$	$0.00x10^{0}$	$1.00x10^{1}$	$0.00x10^{0}$	$2.50x10^{1}$	$7.07x10^{0}$	$3.00x10^{1}$	$1.41x10^{1}$
	05.05.	M	$6.45x10^{2}$	$1.34x10^{2}$	$4.00x10^{1}$	$1.41x10^{1}$	$2.50x10^{1}$	$7.07x10^{0}$	$1.50x10^{1}$	$7.07x10^{0}$	$0.00x10^{0}$	$0.00x10^{0}$	$0.00x10^{0}$	$0.00x10^{0}$	$0.00x10^{0}$	$0.00x10^{0}$	$2.50x10^{1}$	$7.07x10^{0}$	$2.15x10^{2}$	$3.54x10^{1}$
	2022	A										nt								
**F**	12.05.	M	$5.35x10^{2}$	$7.07x10^{0}$	$1.50x10^{1}$	$7.07x10^{0}$	$0.00x10^{0}$	$0.00x10^{0}$	$4.00x10^{1}$	$1.41x10^{1}$	$5.00x10^{0}$	$7.07x10^{0}$	$0.00x10^{0}$	$0.00x10^{0}$	$2.00x10^{1}$	$1.41x10^{1}$	$5.80x10^{2}$	$0.00x10^{0}$	$2.95x10^{2}$	$9.19x10^{1}$
	2022	A										nt								
	19.05.	M	$1.22x10^{3}$	$1.63x10^{2}$	$2.50x10^{1}$	$7.07x10^{0}$	$6.50x10^{1}$	$7.07x10^{0}$	$0.00x10^{0}$	$0.00x10^{0}$	$5.00x10^{0}$	$7.07x10^{0}$	$1.50x10^{1}$	$7.07x10^{0}$	$2.95x10^{2}$	$2.12x10^{1}$	$2.95x10^{2}$	$2.12x10^{1}$	$2.95x10^{2}$	$2.12x10^{1}$
	2022	A										nt								

Location: A - climbing wall; B - swimming pool; C - changing room at the swimming pool; D - basketball / volleyball court; E - badminton court; F - atmospheric air in front of Zatoka Sportu building; Time: M - morning; A – afternoon; Time: M - morning; A – afternoon; M – mean; SD- standard deviation; nt – not tested
